# Polyester and Epoxy Resins with Increased Thermal Conductivity and Reduced Surface Resistivity for Applications in Explosion-Proof Enclosures of Electrical Devices

**DOI:** 10.3390/ma15062171

**Published:** 2022-03-15

**Authors:** Małgorzata Szymiczek, Dawid Buła

**Affiliations:** 1 Department of Theoretical and Applied Mechanics, Silesian University of Technology, 44-100 Gliwice, Poland; 2 Ośrodek Pomiarów i Automatyki S.A., 41-800 Zabrze, Poland; dawid.bula@polsl.pl; 3 Department of Electrical Engineering and Computer Science, Silesian University of Technology, 44-100 Gliwice, Poland

**Keywords:** polymeric composites, thermal conductivity coefficient, surface resistivity, flammability, flexural strength

## Abstract

Composite materials are still finding new applications that require the modification of various properties and are characterized by the summary impact on selected operational features. Due to the operating conditions of electrical equipment enclosures in potentially explosive atmospheres, the surface resistivity ensuring anti-electrostatic properties, i.e., below 10^9^ Ω and resistance to the flame while maintaining appropriate operational enclosure properties is very important. It is also crucial to dissipate heat while reducing weight. Currently metal or cast-iron enclosures are used for various types of electrical devices. As part of the work, a material that can be used for a composite matrix for the enclosure was developed. The study aimed to assess the influence of selected fillers and chemical modifications on the thermal conductivity coefficient, resistivity, and strength properties of matrix materials for the production of electrical device enclosures used in the mining industry. Selected resins were modified with graphite, copper, and carbon black. Tests were carried out on the coefficient of thermal conductivity, surface resistivity, flammability, and flexural strength. At the final stage of the work, a multi-criteria analysis was carried out, which allowed the selection of a composite that meets the assumed characteristics to the highest degree. It is a vinyl ester composite modified with 15 wt.% MG394 and 5 wt.% MG1596 graphite (W2). The thermal conductivity of composite W2 is 5.64 W/mK, the surface resistivity is 5.2 × 10^3^ Ω, the flexural strength is 50.61 MPa, and the flammability class is V0.

## 1. Introduction

The target market for the considered explosion-proof enclosures of electrical devices is primarily the mining industry. Therefore, currently produced casings (steel and cast iron) are subject to specific requirements that are defined by appropriate standards. This is, inter alia, the standard IEC 60079-1 [1], which includes detailed requirements for the construction and testing of electrical equipment intended for use in gaseous explosive atmospheres. In the context of new materials, the most important factors are thermal conductivity and mechanical strength. For example, durability tests of flameproof joints of enclosures are carried out at a pressure of 1.8 MPa (1.5 times the reference pressure of 1.2 MPa). In addition, in mining excavations, plastic products that meet the requirements of flame-retardant and anti-electrostatic properties should be used. The appearance of a spark caused by electrostatic phenomena on the common housing may cause an explosion. Another important aspect is heat dissipation. Each operating electrical device produces heat, and the use of methods (ventilator, liquid cooling) other than natural convection through the housing in hazardous conditions is very problematic. Therefore, it must be possible to dissipate heat by ensuring an appropriate coefficient of the thermal conductivity of the enclosures.

The use of polymeric materials in the mining industry requires specific electrical, thermal (the highest possible coefficient of thermal conductivity), strength, and flammability properties at the V0 level according to UL94 (Standard for Safety of Flammability of Plastic Materials for Parts in Devices and Appliances). For enclosures of electrical devices, the highest possible coefficient of thermal conductivity and electrical surface resistivity (below 10^9^ Ω) are of particular importance. In addition, there are explosive hazards here. It is assumed that such enclosures will be operated (considering the properties of the explosive atmosphere) at a temperature of −20 °C to +60 °C, a pressure of 80 kPa (0.8 bar) to 110 kPa (1.1 bar), and in air with a normal oxygen content, usually 21% by volume [2].

Analyzing the requirements for polymeric materials in such applications, it is reasonable to use polymer composites, in which the matrix will be characterized by high thermal conductivity and surface resistivity. This is important due to the fact that, for example, in high-current junction boxes, a large amount of heat is released (according to the heat balance 50–200 W, depending on the type of box) which must be dissipated outside, and heat transfer in the conditions of a mine is difficult. The literature describes many possible modifications of polymeric materials with both chemical compounds and inorganic fillers. However, in this type of application, the synergistic impact of the introduced modifications, which also affect the mechanical properties, is important.

In case of K. Dai’s [3] research, the use of polyester resin compounds (1-oxo-2,6,7-trioxa-1-phosphabicyclo[2.2.2]octane-methyl diallyl phosphate (PDAP)) to increase the fire resistivity allowed for a considerable reduction in the ability to give off heat and an increase in the oxygen index and the char yield after combustion. Phosphorus compounds have a significant impact on the formation of charring [4]. Kicko-Walczak [5] investigated the effect of ZnSnO_3_ (ZS), ZnS(OH)_6_ (ZHS), Al(OH)_3_, or Mg(OH)_2_ and Sb_2_O_3_ on reducing the flammability of polyester resins. Among the tested compounds, the best flame retardants with the lowest smoke production were ZS and ZHS, while a significant reduction in smoke production was also observed in the case of Sb_2_O_3_.

Kaynak et al. [6] introduced montmorillonite into epoxy resin, which allowed fracture toughness and oxygen index to be improved. The best results were obtained for a 2% filling degree. Rahateker [7] used a synergistic combination of nanotubes and montmorillonites to retard epoxy resins.

In addition to fire resistivity, an important feature is proper heat dissipation, which is inextricably linked with thermal conductivity. For this purpose, the resins are modified, for example, with silicon carbide [8], graphite in various forms [9,10,11], aluminum nitride [12], aluminum oxide [13], copper oxide [14], or boron nitride [13,14,15]. A separate issue is the modification of electrical properties by the use of carbon black and graphite [10,16]. The introduction of fillers also affects the strength properties. It depends on the type and geometry of particles, filler content, and its distribution in the volume [17].

One of the basic criteria adopted during the modification was the possibility of simple and repeatable application in production conditions.

The work’s purpose was to evaluate the influence of selected fillers and chemical modification on the thermal conductivity coefficient, resistivity, and strength properties of matrix materials for the production of electrical equipment enclosures used in the mining industry. Ultimately, it is planned to use the developed materials to significantly reduce the weight of such enclosures.

## 2. Materials for Research and Methodology

**Figure 1:**
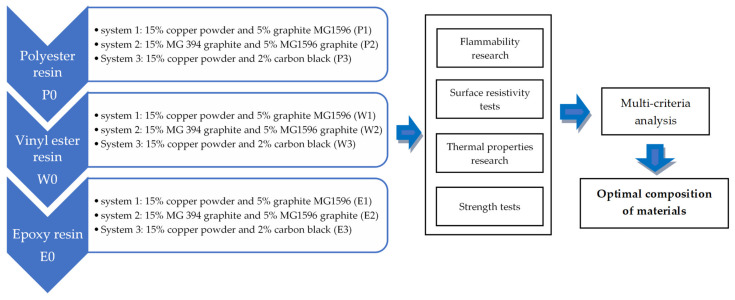
Scheme of the research program.

In the first stage, the assumed aim of the work required the selection of matrix materials and fillers influencing thermal conductivity and surface resistivity. Both a procedure for modifying the matrix material as well as a method for functionalizing the fillers were developed. Moreover, chemical compounds allowing for the improvement of the adhesion, degree of dispersion, and fire resistivity were indicated. The research program is presented in Figure 1 (percentage of filler contents was given by weight).

### 2.1. Material for Research

The tests were carried out on flame retardant resins or resins with increased thermal resistivity:

- Polyester with reduced flammability by the addition of mineral fillers (POLIMAL 1604 TS) cured with a system of 0.4% cobalt accelerator (1% concentration) and 2% LUPAROX K-15, produced by Sarzyna Chemical, Nowa Sarzyna, Poland.
- Vinyl ester (POLIMAL VE-11 MAT) based on brominated bisphenol-A, cured with a system of 1% cobalt accelerator (1% concentration) and 2% low-reactive methyl ethyl ketone peroxide (LUPAROX K-12G), produced by Sarzyna Chemical, Nowa Sarzyna, Poland.
- Epoxy (LG 420 FR from GRM Composites, Olomouc, Czech Republic) with a reduced flammability and an increased resistance to fire, cured with an amine cycloaliphatic hardener with a low viscosity (HG 400) in the ratio 100:25 parts. In addition, the resin was modified in terms of reducing flammability with diethyl-N,N-bis (2-hydroxyethyl)-aminomethyl phosphonate, as well as an agent improving the wettability of the fibers, which facilitates degassing of the resin. Table 1 presents the selected properties of the tested resins.

**Figure 2:**
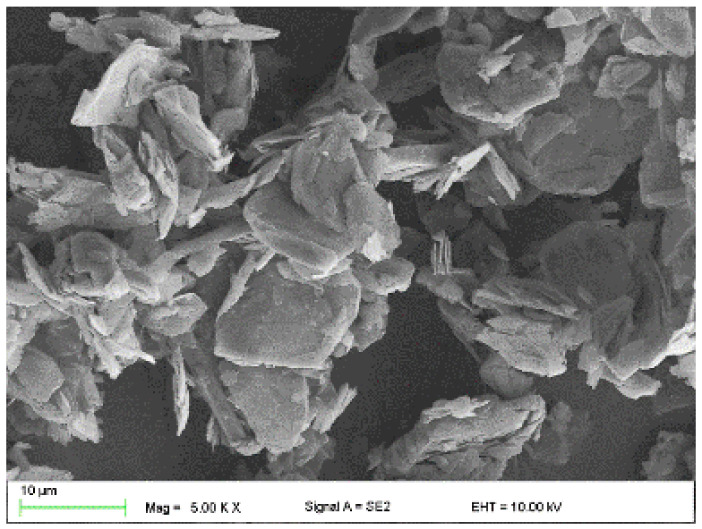
Graphite MG394.

**Figure 3:**
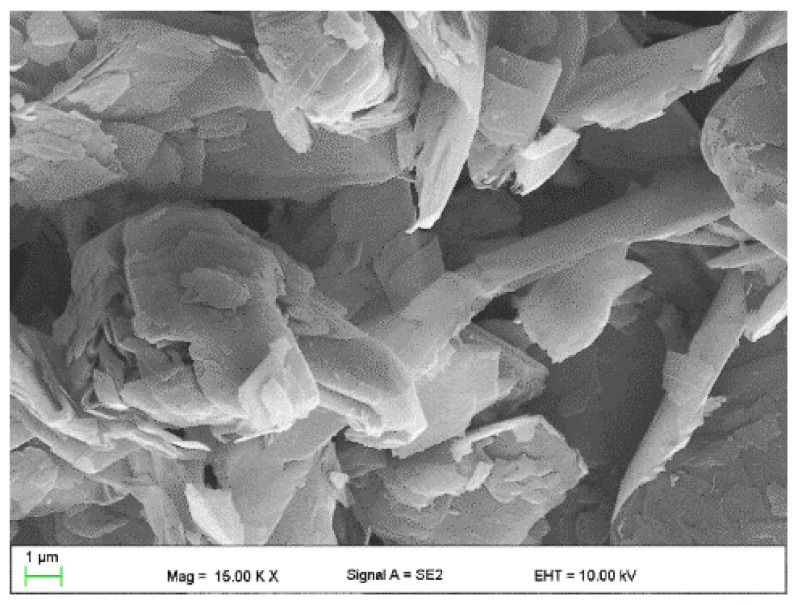
Graphite MG1596.

**Figure 4:**
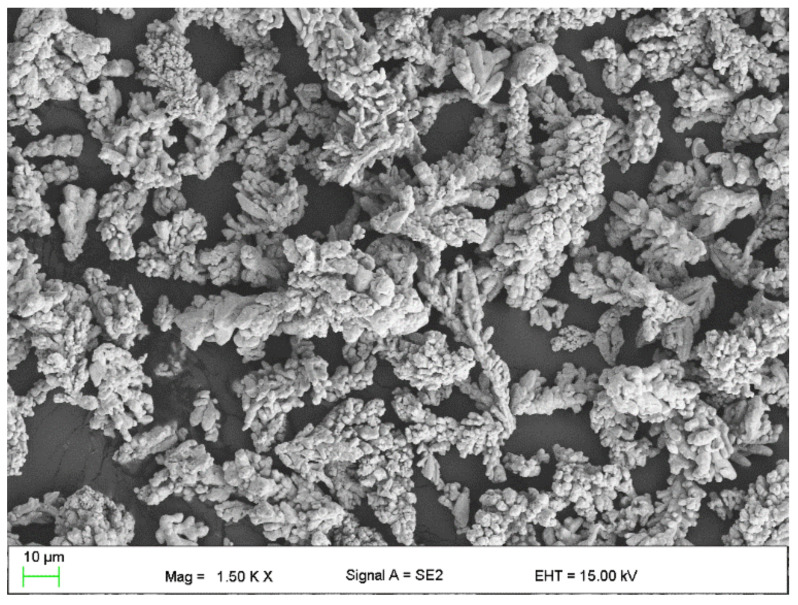
Copper powder LT16.

The following were used to modify the resins:

- Flake graphite MG 394 (grain size (flake) < 45 µm) and MG 1596 (grain size (flake) < 10 µm) (Sinograf, Toruń, Poland) (Figure 2 and Figure 3) with a thermal conductivity from 140 to 233 W/mK depending on the character.
- Copper powder (Lt16 from Stanchem, Niemice, Poland) with a dendritic particle shape and grain size from 150 to 32 µm (Figure 4) with a thermal conductivity of 380 W/mK.

**Figure 5:**
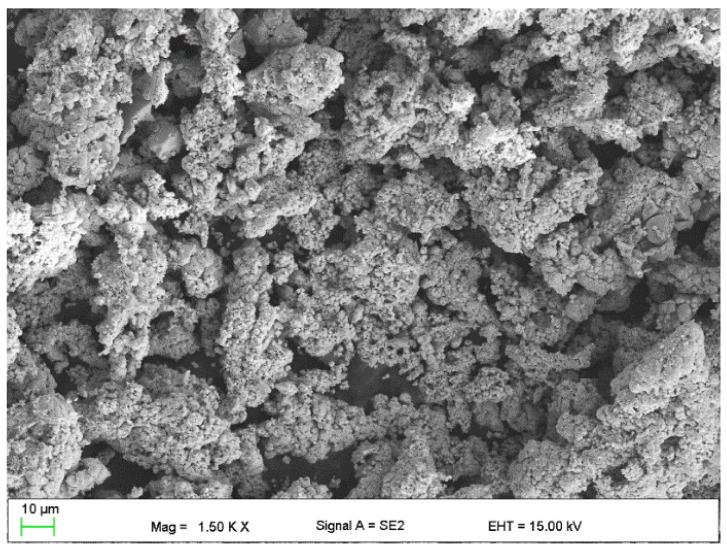
Copper powder after etching.

To remove copper oxides from the surface and to ensure proper wetting of the filler by the matrix material, a copper etching process was carried out in a solution of 40 g of 2-hydroxy-1,2,3-propane tricarboxylic acid, 20 g of sodium chloride (NaCl), and 200 mL of a 3% hydrogen peroxide solution (H_2_O_2_). The etching time was 5 h. Then, the filler was filtered and rinsed with demineralized water until it was transparent. The view of the etched dendritic copper is shown in Figure 5. The morphology of the fillers was visualized using a Zeiss Supra 35 scanning electron microscope (Carl Zeiss AG, Oberkochen, Baden-Württemberg, Germany).

The surface resistivity was modified by the use of MG1596 graphite and carbon black FW 200 (Degussa Goldhandel GmbH, Munich, Germany), which for many years has been used in the mining industry as a filler to reduce surface and volume resistivity in polymer composites (e.g., in ventilation pipes, conveyor belts) [21]. The used carbon black had an average grain size of 13 μm, a density of 1.7–1.9 g/cm^3^, and a pH of 6.5 [22].

Selected fillers, before being introduced into the matrix, were subjected to surface treatment to ensure an appropriate adhesive join. Functionalization was carried out by the interaction of the solution: demineralized water-ethyl alcohol in the ratio of 25:75 with 3% of the appropriate silanes produced by Unisil, Tarnów, Poland. U-611 (vinyl trimethoxysilane-C_5_H_12_O_3_Si) with a molecular weight of 148.2 g/mol was used for polyester and vinyl ester resins. The epoxy resin was modified with U-15 (N-2-aminoethyl-3-aminopropyltrimethoxysilane-C_8_H_22_N_2_O_3_Si) with a molecular weight of 226.36 g/mol.

Then, a 10 g filler was introduced per 1000 mL and mixed for 1 h at a speed of 3000 rpm on a High-Speed Dissolver Dispermat LC30 mixer (VMA-Getzmann GMBH, Reichshof, Germany). The solution was stayed for 48 h at 25 ± 2 °C, filtered, rinsed with demineralized water, and dried in a vacuum dryer for 8 h at 80 °C. Prepared fillers were introduced into the resin by the mixing method on a High-Speed Dissolver Dispermat LC30 mixer equipped with discs (diameter of 50 mm) with a mixing speed of 4000 rpm for 1 h, then cooled in a water bath to 25 °C; a curing system was introduced and re-mixed for 5 min at a speed of 1000 rpm.

In the case of the POLIMAL VE-11 MAT vinyl ester resin (Organika Sarzyna, Sarzyna, Poland), 2% of the tertiary amine was added to modify the curing system. The use of cobalt + amine systems allows the gel and curing time to be controlled, which extends the working conditions. The resulting compositions were vented in a vacuum dryer with a vacuum of 0.08 MPa at 20 ± 2 °C (room temperature). The fillers were modified based on the procedures described in [9,10].

The samples were made into silicone molds by gravity casting. After cross-linking, the samples were ground to ensure the parallelism of the surface. Before the implementation of the assumed test program, the samples were conditioned in forced air dryers at a temperature of 80 ± 2 °C for 4 h. This is the first stage of work, which consists of the appropriate preparation of modified resins which will be used as the matrix material in the composite enclosures of electrical equipment used in the mining industry.

**Figure 6:**
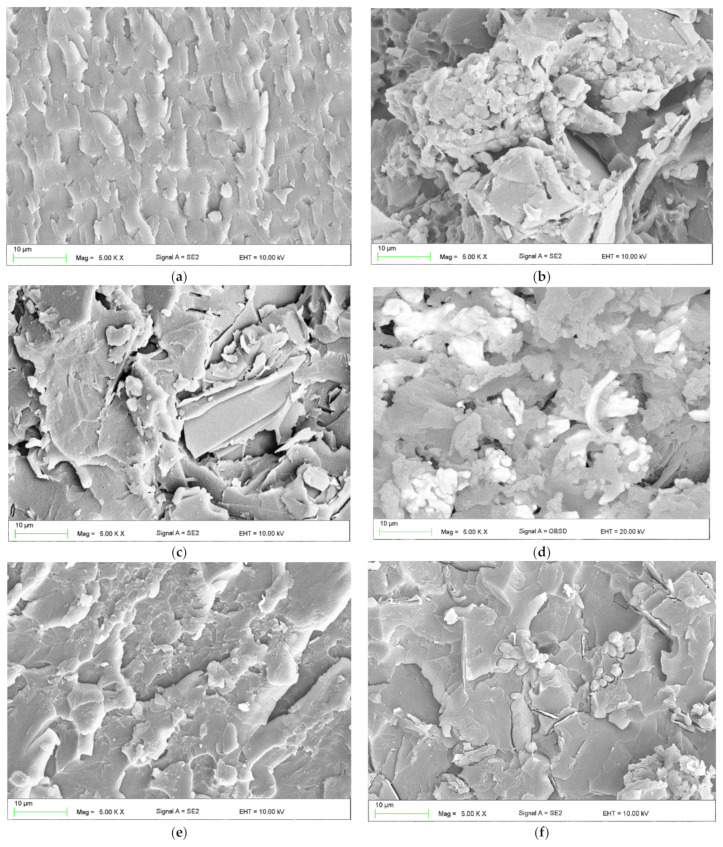

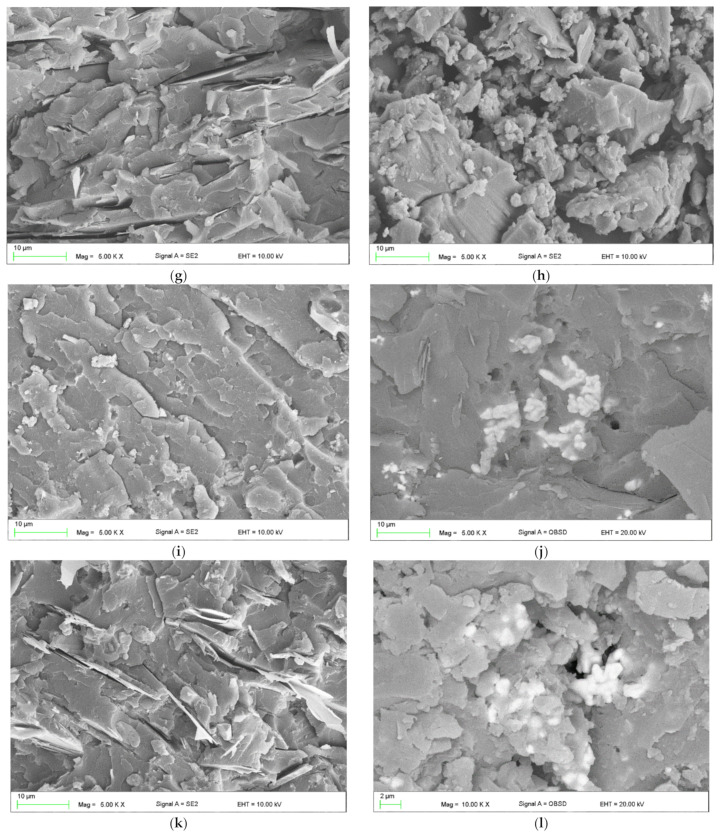
SEM images of tested materials: (**a**) E0, (**b**) E1, (**c**) E2, (**d**) E3, (**e**) W0, (**f**) W1, (**g**) -W2, (**h**) W2, (**i**) P0, (**j**) P1, (**k**) P2, (**l**) P3.

Figure 6 shows micro photos of the structure taken with the SEM microscope. The samples were sprayed with a layer of gold for 90 s. The fracture surfaces after flexural were tested. In the first part of the study, the SE (secondary electron) signal was carried out (Figure 6). However, due to the lack of possibility to observe dendritic copper, backscattered electron (BSE) imaging was used.

As can be seen, the best homogenization was obtained for composites filled with graphite, which were characterized by high operational properties and a low standard deviation. This is undoubtedly related to the shaping of the internal structure. In the case of composites E3, W3, and P3, it was observed that the copper particles were well wetted with resin and soot, which made the analysis difficult. There was a slight sedimentation of the copper particles. Hence, the results of the thermal and strength tests for the samples modified with copper are characterized by a greater dispersion of the results. The slight sedimentation of copper particles could influence the surface resistivity measurement. However, the difference in the measurement of the surface resistance on the two opposite surfaces was about 8%. A similar effect was observed for composites E1, W1, and P1, but to a lesser extent, which is related to, e.g., the particle size of the graphite used.

### 2.2. Thermal Properties Research

The thermal conductivity coefficient was determined based on the measurement of specific heat, density, and thermal diffusivity.

Thermal diffusivity tests were carried out on the described stand [23], where the thermal pulse is generated by an infrared radiator, and the temperature distribution on the surface is registered with an FLIR A615 camera (FLIR Systems, Inc, Wilsonville, OR, USA). The stand ensures homogeneous heating conditions by maintaining a constant distance of the infrared heater from the heated sample. The FLIR A-615 thermal imaging camera, cooperating with IRControl software (FLIR Systems, Inc, Wilsonville, OR, USA), was used to record temperature changes. The samples with dimensions of 200 mm × 20 mm× 10 mm were heated for 30 ± 2 s. Thermal diffusivity, defined as the time course of temperature on the surface opposite to the thermally activated surface, was determined by this method. It does not take into account heat exchange with the environment or climatic conditions, and the thermal activation time is assumed to be infinitely short. The diffusivity was determined from the following relationship [24]:

(1) $$d_{c}=1.38\frac{g^{2}}{\pi^{2}t_{1/2}}$$

where g is the thickness (mm) and t_1/2_ is half the time to maximum temperature in seconds.

The density of the prepared composites was tested on an analytical balance (Ohaus Adventurer Pro, OHAUS Europe GmbH-Nänikon, Greifensee, Switzerland) equipped with a hydrostatic density measurement set, following ISO 1183-1 [25]. The test consisted of measuring the mass of the sample in the air, and then in demineralized water of known density (ρ_w_ = 0.998 g/cm^3^). Based on the obtained results, the density ρ (g/cm^3^) of the samples was determined by the following formula:

(2) $$\rho =\rho_{w}\frac{m_{1}}{m_{1}-m_{2}},$$

where *m*_1_ and *m*_2_ are the sample mass in air and demineralized water, respectively (g). The density value is the average of five measurements. The tests were carried out at a temperature of 20 ± 2 °C and a humidity of 50 ± 5%.

The specific heat was determined by the differential scanning calorimetry method by ISO 11357-4: 2013 [26] using the DSC 1 Star System apparatus by Mettler Toledo (Columbus, OH, USA). Specially prepared samples with a diameter of 12.5 mm and a thickness of 2.5 mm were used for the tests.

Based on the determined thermal diffusivity, density, and specific heat, the thermal conductivity was calculated from the following formula:

(3) $$\lambda =d_{c}c_{p}\rho ,$$

where *c_p_* is the specific heat (J/kg K) and ρ is the density (g/cm^3^).

The developed methodology of testing the thermal conductivity coefficient can be used without limiting the thickness of the samples.

### 2.3. Surface Resistivity

**Figure 7:**
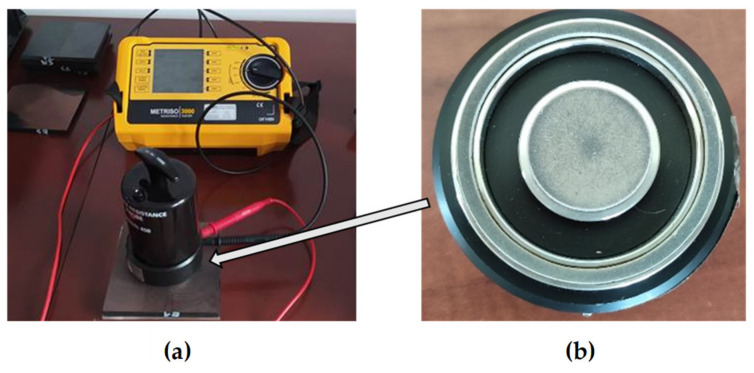
Electrical test stand (**a**) with measuring head (**b**).

The test was carried out by IEC 61340-2-3 [27] on a Metriso 3000 m (Wolfgang Warmbier, Hilzingen, Germany) which was designed for the surface/volume and between points resistivity. The tests were carried out on the measuring system shown in Figure 7a, consisting of a meter and ring electrodes with a diameter of 63 mm (Figure 7b) for measuring the surface resistivity. A model 880 ring electrode and model 850 electrodes were used for a contact pressure of 2.5 kg (IEC 61340-2-3).

The research was conducted under the following conditions:

- A 10 V voltage for results below 10^6^ Ω and 100 V for results above 10^6^ Ω;
- A measurement time of 15 s by the recommendations of the IEC 61340-2-3 standard;
- A test temperature of 20 °C and a humidity of 60%.

**Figure 8:**
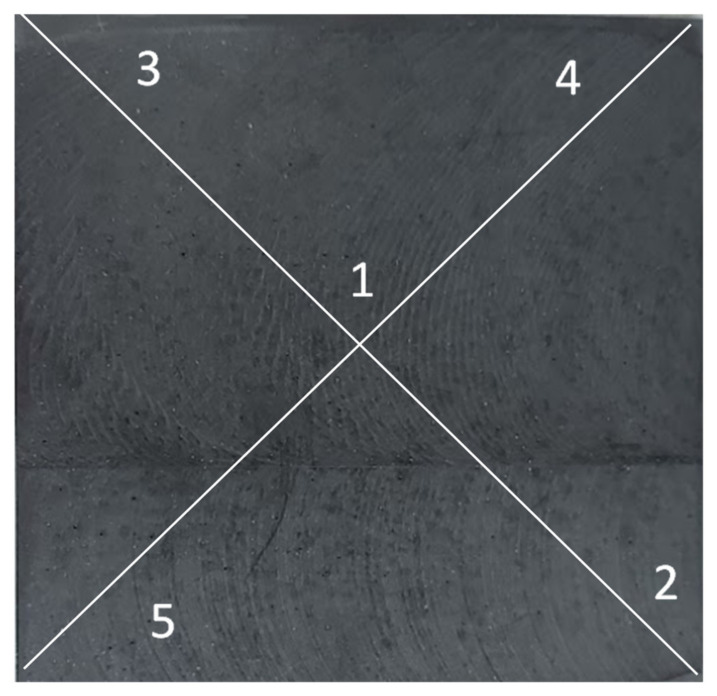
View of the sample prepared for testing.

The tests were carried out on samples with dimensions of 120 mm × 120 mm × 10 mm with the outer matrix layer removed (Figure 7). The outer layers were removed by milling and then sanded with P180 sandpaper. The samples were prepared with the parallelism of the surface. It is especially important to remove from the surface blisters that interfere with the measurement in a significant way. Before testing, all samples were conditioned for 48 h at 20 °C, and then at 60 °C for 2 h, and degreased with ethanol. For each sample, five measurements were made by the diagram shown in Figure 8.

### 2.4. Flammability Tests

As a part of the tests, the class of flammability and fire resistance was determined. Determination of the flammability category was carried out on samples with dimensions of 125 mm × 13 mm × 10 mm. The samples were conditioned at 23 ± 2 °C and 50 ± 5% relative humidity for 48 h; the second set of samples was conditioned at 70 ± 1 °C for 168 h. The tests were carried out by the EN 60695-11-10: 2014-02 method B [28] standard, with a 50 W Bunsen burner, fueled with 98% pure industrial methane.

Fire resistance was determined by ISO 340: 2013-07 [29], taking into account ISO/IEC 80079-38: 2017-02 p.6.2 [30] using a Bunsen gas burner. Samples with dimensions of 200 mm × 25 mm × 10 mm were tested. Each sample was set on fire for 10 s and the burning time was measured after the removal of the burner. Within 60 ± 5 s from the end of the burning time, an airstream was directed at the sample perpendicular to its surface for the 60 s. The samples were conditioned at 23 ± 2 °C and 50 ± 5% relative humidity for 48 h. The tests were carried out at the temperature of 22 ± 2 °C, humidity of 55%, and atmospheric pressure of 980 hPa. Only polymer resins, which were the matrix of the composite, were tested. According to the literature, it was assumed that the introduced fillers would not affect the flammability class and fire resistance [31].

### 2.5. Flexural Strength Tests

Flexural strength tests were carried out by ISO 178 [32], on samples with dimensions of 80 mm × 10 mm × 4 mm. The tests were carried out on a Shimadzu AGX-10 kN D testing machine (Shimadzu Corporation, Kyoto, Japan) cooperating with the Trapezium X software (Shimadzu Corporation, Kyoto, Japan) and appointed with appropriate equipment for three-point bending. The spacing of the supports was 64 mm. The radius of the supports was 5 mm. The tests were carried out at a speed of 2 mm/min. The ambient temperature was 20 ± 2 °C, and the humidity was 60%. The flexural strength and flexural modulus were determined.

## 3. Results and Discussion

For the presented materials, research was carried out according to the methodology presented above.

### 3.1. Thermal Properties Results

**Figure 9:**
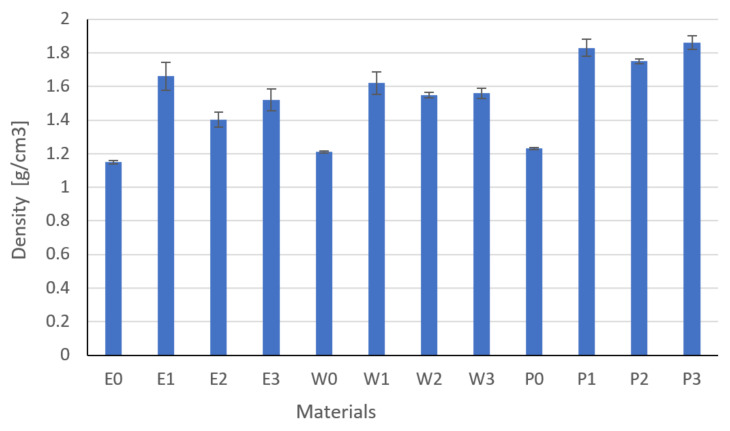
Results of density.

**Figure 10:**
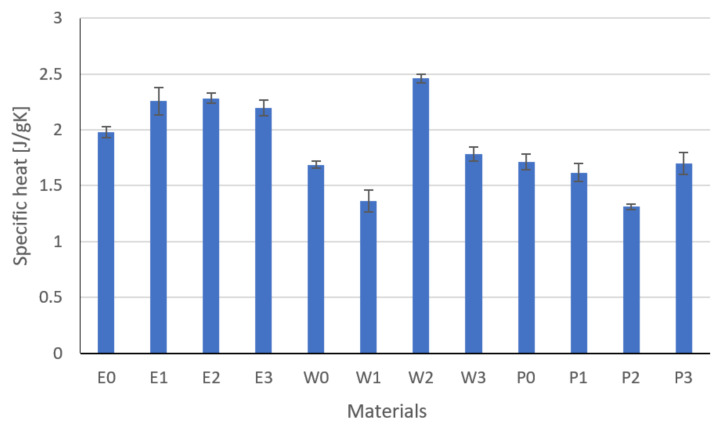
Results of specific heat.

**Figure 11:**
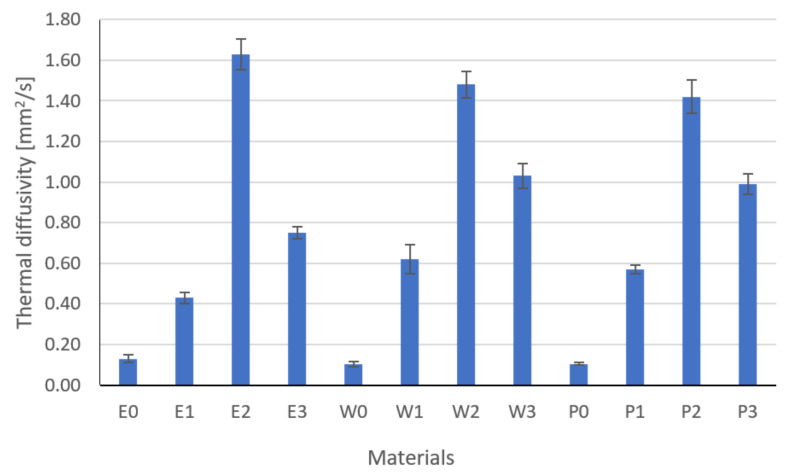
Results of thermal diffusivity.

**Figure 12:**
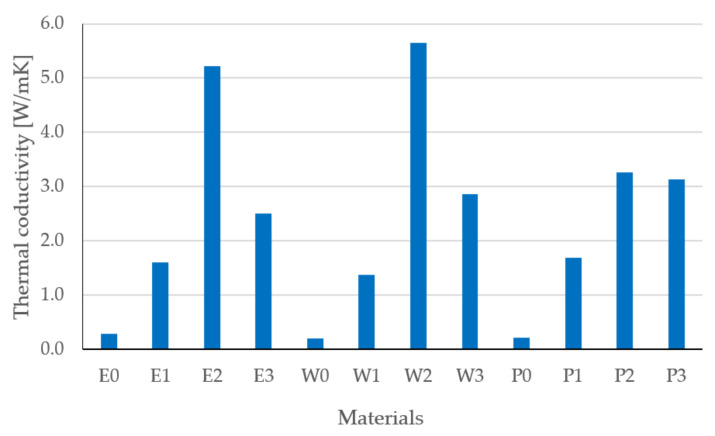
Results of thermal conductivity coefficient.

Based on the measurements of density (Figure 9), specific heat (Figure 10), and thermal diffusivity (Figure 11), the thermal conductivity was calculated by Equation (3) (Figure 12).

All composites showed higher values than the unmodified resins, but the greatest changes were noted for composites filled with copper particles. In the case of epoxy resins, copper sedimentation was observed, which is related to its low viscosity and filler density. The smallest changes in density were observed for composites filled with graphite particles. The highest standard deviation was noted for epoxy composites, and the lowest for W2 and P2 composites. Among all materials, the samples filled with copper and graphite showed the highest standard deviation. This is related to distributing the filler throughout the sample and preparing the sample. The observed differences, especially in the case of epoxy resins, may be related to the surface tension and preparation of samples (grinding to maintain the parallelism of the surface).

The data analysis showed that the type of filler and its content had a significant effect on the density (Fisher statistic F = 95.07, critical F = 2.21).

The highest specific heat was characteristic of composites filled with E2 and W2 modified graphite. In the case of epoxy matrix composites, all composites showed higher specific heat values related to E0. The differences were 11–15%. Samples with a vinyl ester matrix showed a lower specific heat for the sample W1, which may have been related to the insulating compounds and appropriate distribution of the filler in the matrix. W2 had the highest specific heat of 2.46 J/gK. Composites with a polyester matrix were characterized by the lowest specific heat, which was related to the flame retardation of the resin with mineral fillers.

The presented results of diffusivity (Figure 11) are the average of 10 measurements. Differences in the temperature measurement on the sample surface were observed, which confirms the uneven distribution of fillers in the material. The greatest differences, approximately 20%, were observed for composites with an E1 epoxy matrix and P2 polyester matrix. The conducted analysis showed that the type of the filler had a significant influence on the specific heat (Fisher statistic F = 1040.46, critical F = 1.88). The lowest thermal diffusivity was shown by composites filled with copper powder and graphite (E1, W1, P1). The composites filled with graphite E2, W2, and P2 were characterized by the highest diffusivity and the smallest standard deviation.

The determined thermal conductivity coefficient for the tested composites is shown in Figure 12. As can be seen, the highest thermal conductivity was observed for the composites E2, W2, and P2. The composite W2 had the highest thermal conductivity coefficient of 5.64 W/mK. The calculated coefficient is the result of measurements of density, specific heat, and diffusivity; therefore, the obtained value is subject to some error.

### 3.2. Surface Resistivity Results

**Figure 13:**
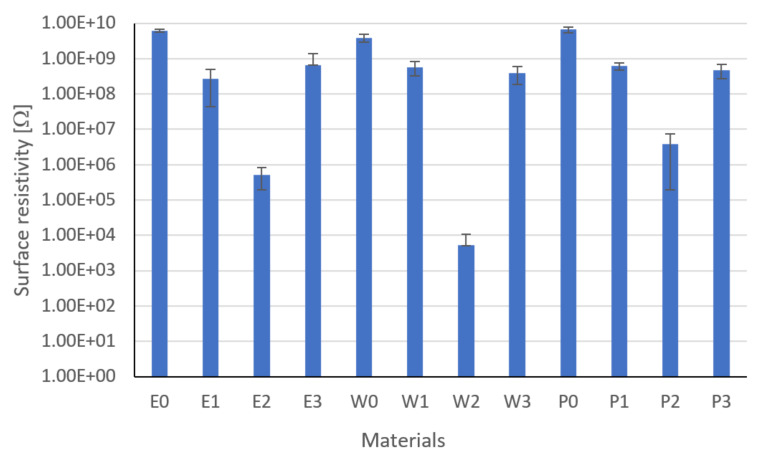
Surface resistivity results.

Figure 13 shows the results of the surface resistivity. The results shown are the mean of five measurements. The lowest values were characteristic for composites filled with graphite—E2, W2, and P2—of which the lowest value is characterized by W2: 5.19 × 10^3^ Ω. At the same time, W2 and P2 are characterized by the smallest standard deviations. The applied modifications (graphite and carbon black) lowered the resistivity concerning unmodified resins. Statistical analysis showed that the type of filler influences the surface resistance value (Fisher statistic F = 96.04, critical F = 1.95). The highest standard deviations were observed for the epoxy matrix composites, which is related to the internal structure and viscosity of the resin.

### 3.3. Flammability Results

Table 2 shows the results of flammability tests according to EN 60695-11-10: 2014-02 method B. The presented values are the average of five measurements.

As can be seen, the lowest values of all times were observed for the polyester resin flame retarded with mineral fillers. The vinyl ester resin, for which the V0 flammability class was also determined, shows 1.5–2 times longer burning and glow times. The epoxy resin, for which the flammability class has not been determined, shows the longest times, despite the use of modifiers, which improved fire resistance and were used to improve wettability.

In the case of determining fire resistance, it is also the polyester resin which shows the lowest maximum value of burning time: 1.3 s. The maximum burning time specified for vinyl ester resin is 3.5 s, while for epoxy resin it is over 20 s (to be burned) despite the modification applied.

### 3.4. Flexural Strength Results

**Figure 14:**
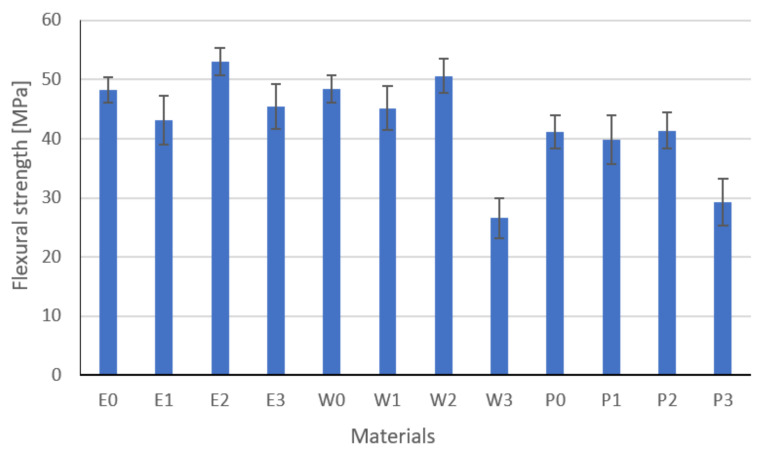
Flexural strength results.

**Figure 15:**
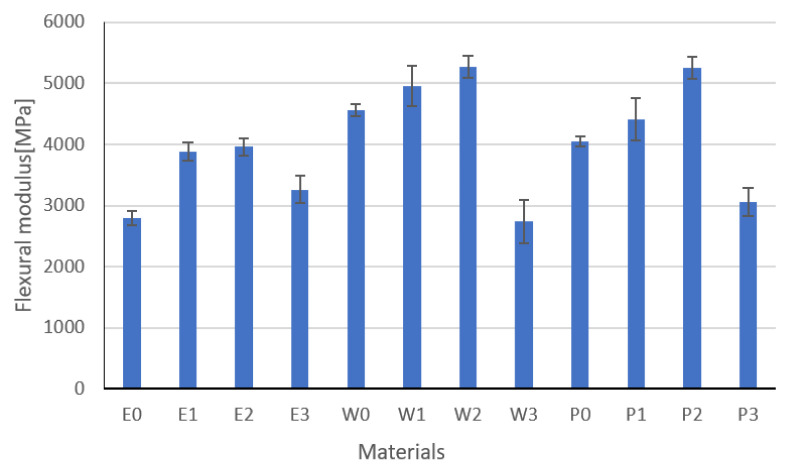
Flexural modulus results.

**Figure 16:**
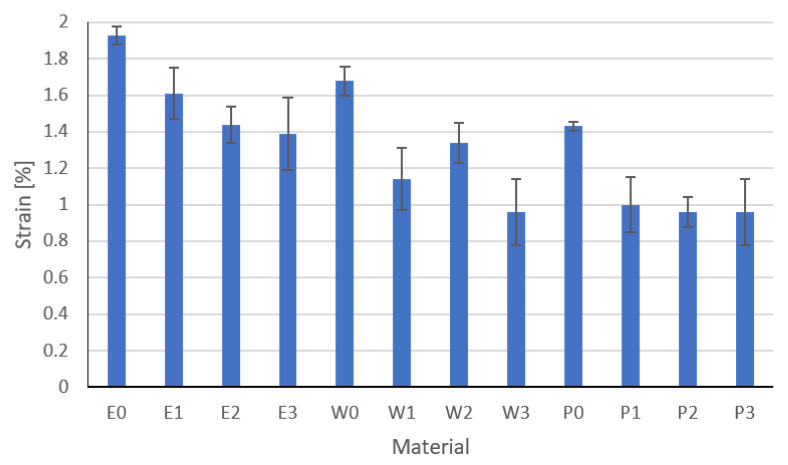
Strain at break results.

Results of the flexural strength are, modulus, and strain at break are shown in Figure 14, Figure 15 and Figure 16, respectively. These are the average results from five measurements. In the event of a large discrepancy in the obtained results, the test was repeated.

Based on the analysis of the results, it was found that the fillers change the strength properties. The highest values of flexural strength are characteristic for resins modified with functionalized graphite. The highest values of flexural strength were recorded for epoxy resin E2. The greatest strain was recorded for the E0 resin, which is related to the flame retardant system used. The W2 vinyl ester resin has a flexural strength similar to that of the epoxy resin and the highest modulus of elasticity. The highest standard deviations were recorded for composites modified with copper powder with graphite or carbon black, while the smallest were for composites modified with graphite powders. This is undoubtedly related to the grain size of the filler and its weight. Inaccurate homogenization can cause the filler particles to act as a notch. The type and content of the filler and the degree of its homogenization have a significant impact on the tested parameters. In the case of copper, significant sedimentation of the filler was observed, especially in the epoxy resin, which is related to its viscosity. In addition, sedimentation causes the formation of layers that, due to the different stiffnesses, are characterized by another cracking mechanism. Parts with a higher modulus of elasticity break first. The conducted one-way analysis of variance showed, as in the previous studies, that the type of filler has a significant impact on the tested properties (Fisher statistic F = 7664.24, critical F = 5.31).

### 3.5. Multi-Criteria Analysis

The results of the conducted research were the basis for a multi-criteria analysis. Criteria for analysis were developed based on the requirements for explosion-proof enclosures of electrical equipment operating in the mining industry. The criteria were given weights from 0 to 5. The adopted scoring system corresponded to the requirements for polymeric materials used in the mining industry. For such applications, surface resistivity and fire resistance are of major importance. Flammability was set at level 1 because it was assumed that it is a superior characteristic. Elements approved for use in mining must show the V0 flammability class. Minimal differences between the vinyl ester and polyester resins are due to the burning times. However, due to the application, the most important factor is the thermal conductivity coefficient, which has a significant influence on the heat dissipation from the inside of the enclosure. Next, the strength properties were important. The proposed weights were the result of analyses carried out using heuristic techniques (the Delphi method). Tested materials were evaluated from 1 to 12, with a rating of 0 for the undefined flammability class. In the case of the thermal conductivity coefficient for unmodified materials, a rating of 1 was adopted, because the differences between them were within the error limits. Table 3 shows the results of the multi-criteria analysis. The highest properties were characteristic of resins modified with graphite particles in the order W2 (176 points), E2 (147 points), P0 (139 points).

## 4. Conclusions

Based on the conducted research, the following was concluded:

- The type and content of the filler had a significant impact on the thermal conductivity, resistivity, and the tested strength properties, which was confirmed by the one-way analysis of variance.
- Modification and the developed technology of introducing fillers into the resin allowed for the production of composites with increased thermal conductivity and lowered resistance. The highest coefficient of thermal conductivity was observed for composites E2, W2, and P2 filled with MG394 and MG1596 flak graphite. The highest resistance was also noted for the materials mentioned. This is the result of the fillers being evenly distributed over the cross-section.
- The lowest properties concerning graphite-modified composites were found in composites filled with copper powder and technical carbon black. This is a result of the uneven distribution of the filler on the cross-section and the phase separation (copper sedimentation).
- The highest values of flexural strength were also shown by graphite-modified composites, which confirms the results of thermal conductivity and resistance.
- The best summary effect of improving the tested properties was presented by composite W2 (176 points in the multi-criteria analysis).
- The developed W2 composite (MG394 and MG1596 graphite and vinyl ester resin) can be used as a matrix material for the production of laminates that are enclosures for electrical devices operating in explosive conditions.

## Figures and Tables

**Table 1:** Properties of resins [18,19,20].

Property	Standard	Unit	Polyester Resin	Vinyl Ester Resin	Epoxy Resin
Viscosity at 25 °C Brookfield	ISO 3219	mPa s	550–650	300–400	900–1200
Gel time at 25 °C	ISO 2535	min	34	30	3 h 17 min
Deflection temperature HDT	ISO 75	°C	94	90	-
Flammability class	EN 60695-11-10:2014-02	-	V0	V0	Undefined
Compressive strength	ISO 604	MPa	120	126	132
Flexural strength	ISO 178	MPa	90	130	120
Tensile strength	ISO 527	MPa	67	80	72
Young’s modulus	ISO 527	MPa	3700	3600	3300
Strain at break	ISO 527	%	2.3	3.5	6
Barcol hardness	ASTM D 2583-95 ASTM D 2583	B/Sh D^o^	48	40	85 ^o^

**Table 2:** Flammability results.

Resin Parameter	Epoxy E0	Vinyl Ester W0	Polyester P0
Test conditions	23 ± 2 °C/50 ± 5%	70 ± 2 °C	23 ± 2 °C/50 ± 5%
Burning time after 1st flame application t2 (s)	34.96	39.04	3.002
Burning time after 1st flame application t1 (s)	66.14	65.54	4.88
Glowing time after the second flame application t3 (s)	0	0	0
Total burning time t = S (t1 + t2) (s)	505.5	522.9	36.6
Time of burning and glowing after the second flame application (t2 + t3) (s)	66.14	65.54	4.88
Inflammation of cotton wool, yes/no	no	no	no
Burning, glow to terminals, yes/no	no	no	no
Flammability category	Cannot be categorized	V0—10.6 mm	V0—10.3 mm

**Table 3:** Multi-criteria analysis.

Characteristic	Weights	Materials											
		E0		E1		E2		E3		W0		W1	
		C	W	C	W	C	W	C	W	C	W	C	W
Flammability class	1	-	0	-	0	-	0	-	0	V0	11	V0	11
		0		0		0		0		11		11	
Thermal conductivity coefficient, W/mK	5	0.29	5	1.61	20	5.22	55	2.51	35	0.21	5	1.37	25
		1		4		11		7		1		5	
Surface resistivity, Ω	4	6.1 × 10^9^	8	2.7 × 10^8^	36	5.2 × 10^5^	44	6.5 × 10^8^	24	3.9 × 10^9^	12	5.8 × 10^8^	20
		2		9		11		6		3		5	
Flexural strength, MPa	3	48.2	27	43.1	18	53.01	36	45.45	24	48.43	30	45.19	21
		9		6		12		8		10		7	
Flexural modulus, GPa	2	2.8	4	3.89	10	3.96	12	3.27	8	4.56	18	4.96	20
		2		5		6		4		9		10	
Σ			44		84		147		91		76		97
**Characteristic**	**Weights**	**Materials**											
		**W2**		**W3**		**P0**		**P1**		**P2**		**P3**	
		**C**	**W**	**C**	**W**	**C**	**W**	**C**	**W**	**C**	**W**	**C**	**W**
Flammability class	1	0	11	V0	11	V0	12	V0	12	V0	12	V0	12
		11		11		12		12		12		12	
Thermal conductivity coefficient, W/mK	5	5.64	60	2.86	40	0.21	5	1.69	30	3.25	50	3.13	45
		12		8		1		6		10		9	
Surface resistivity, Ω	4	5.2 × 10^3^	48	3.9 × 10^8^	32	6.7 × 10^9^	4	6.2 × 10^8^	16	3.9 × 10^6^	40	4.7 × 10^8^	28
		12		8		1		4		10		7	
Flexural strength, MPa	3	50.61	33	26.58	3	41.2	12	39.84	9	41.36	15	29.28	2
		11		1		4		3		5		2	
Flexural modulus, GPa	2	5.27	24	2.74	2	4.05	14	4.42	16	5.26	22	3.06	6
		12		1		7		8		11		3	
Σ			176		88		47		83		139		93

C—Criterion assessment, W—value.

## Data Availability

Data sharing is not applicable.
